# Postpartum Women’s Preferences for Lifestyle Intervention after Childbirth: A Multi-Methods Study Using the TIDieR Checklist

**DOI:** 10.3390/nu14204229

**Published:** 2022-10-11

**Authors:** Maureen Makama, Mingling Chen, Lisa J. Moran, Helen Skouteris, Cheryce L. Harrison, Tammie Choi, Siew Lim

**Affiliations:** 1 Monash Centre for Health Research and Implementation, Monash University, Clayton, VIC 3168, Australia; 2 Health and Social Care Unit, School of Public Health and Preventive Medicine, Monash University, Melbourne, VIC 3004, Australia; 3 Warwick Business School, Warwick University, Coventry CV4 7AL, UK; 4 Department of Nutrition, Dietetics, and Food, School of Clinical Sciences, Monash University, Notting Hill, VIC 3168, Australia; 5 Eastern Health Clinical School, Monash University, Box Hill, VIC 3128, Australia

**Keywords:** postpartum women, preferences for lifestyle intervention, TIDieR checklist, multi-methods study

## Abstract

Postpartum lifestyle interventions are known to be efficacious in reducing postpartum weight retention, but uptake and engagement are poor. This multi-method study explored the preferences of postpartum women for the delivery of lifestyle interventions based on the Template for Intervention Description and Replication (TIDieR) checklist. Semi-structured interviews were conducted with 21 women within 2 years of childbirth, recruited through convenience and snowball sampling throughout Australia (15 May 2020 to 20 July 2020). Transcripts were analysed thematically using an open coding approach. A cross-sectional online survey was conducted in November 2021 among postpartum women within 5 years of childbirth in Australia. Data were summarised using descriptive statistics. The survey was completed by 520 women. Both the survey and interviews revealed that women were interested in receiving lifestyle support postpartum and wanted a program delivered by health professionals. They preferred a flexible low-intensity program embedded within existing maternal and child health services that is delivered through both online and face-to-face sessions. Having a pragmatic approach that taught practical strategies and enlists the support of partners, family and peers was important to mothers. Consumer-informed postpartum lifestyle interventions promote optimal engagement and improve program reach and therefore, impact.

## 1. Introduction

Many women gain substantial weight during their childbearing years [1]. An estimated one in five women with a healthy pre-pregnancy body mass index (BMI) develop overweight, while 7.6% develop obesity within 3 years of childbirth [2,3]. Excessive gestational weight gain and postpartum weight retention are the main drivers of overweight and obesity in women of reproductive age [4,5]. On average, postpartum weight retention ranges between 0.5 and 3.0 kg and is highly variable, with up to 20% of women retaining >4 kg by 1 year postpartum [2,6,7]. Postpartum weight retention contributes to visceral obesity and increased risk of lifestyle-related diseases such as diabetes, cardiovascular diseases, and metabolic syndrome [8].

The efficacy of postpartum lifestyle interventions for reducing postpartum weight retention and preventing chronic lifestyle diseases is an emerging research area of increasing importance [9,10]. However, some barriers make this population hard to reach and little evidence exists in real-world pragmatic research [6]. Poor engagement and high attrition are general barriers in intervention trials targeting postpartum women [2,11]. A systematic review reported very low recruitment rates in postpartum interventions, underscoring the challenges of engaging women in this life stage in lifestyle modifications [12]. The postpartum period is a time when women are faced with several barriers that may impede engagement such as time constraints, fatigue, and caregiving responsibilities [13]. Addressing these barriers through ensuring that interventions for postpartum women include components that are acceptable and tailored to their needs will enhance engagement with the program and reduce attrition.

The Template for Intervention Description and Replication (TIDieR) checklist is a tool for describing components of intervention including the what, who, how, where, when, and how much intervention is delivered to allow successful implementation and replication [14]. It is a valuable tool for facilitating the effective implementation of interventions through a comprehensive description and documentation of the intervention which can aid future replication or scale-up [14,15]. Understanding the preferences of the end-users of an intervention program is central to the design and implementation of an effective program. The aim of this study was therefore to explore postpartum women’s preferences for a lifestyle intervention program based on the TIDieR checklist to inform the development of a lifestyle intervention program to reduce postpartum weight retention. To better understand the preferences of women for lifestyle support in the postpartum period, this study used a multi-methods approach to answer the same research question (what are the preferred intervention characteristics of postpartum women based on the TIDieR checklist?), providing both a broad and an in-depth perspective of women [16].

## 2. Materials and Methods

### 2.1. Study Design

This study used a multi-methods design that included two independent data collection processes. First, a 1:1 qualitative descriptive semi-structured interview with postpartum women (birth to 2 years) was conducted. Qualitative data provide an in-depth understanding of the reasons behind women’s intervention preferences. However, to obtain a broader perspective from a larger sample and quantify the preferences, a quantitative survey was conducted to answer the same research question in women who had given birth within the past 5 years. We chose to increase the time since childbirth to 5 years in the survey to increase the sample size and allow for comparisons of intervention preferences between women with children under 2 years and those with children 2–5 years. We hypothesized that there will be variation in the preferred intervention characteristics in women with older compared to those with younger children. The qualitative study was conducted and reported according to the Consolidated Criteria for Reporting Qualitative Research (COREQ) guidelines [17].

The qualitative study was approved by the Monash University Human Research Ethics Committee (HREC) (Project number: 22952) and Monash Health HREC (Reference number: RES-19-0000-685A). The quantitative study was approved by the Monash University HREC (Project number: 29273). Interview participants provided audio-recorded verbal consent while survey participants provided informed consent at the start of the online survey.

### 2.2. Study Participants

Interview participants were recruited through convenience and snowball sampling throughout Australia (15 May 2020 to 20 July 2020). Purposive sampling was also done to ensure representation from rural and remote areas (*n* = 2) and cultural and linguistically diverse backgrounds (by country of birth, *n* = 8). All the interviews were conducted in the English language. Participants were women who had given birth within the past 2 years and were living with the child. Prospective participants were contacted via word-of-mouth by colleagues, friends, and other participants. Due to the nature of the convenience sampling, some of the participants had a prior relationship with the interviewers and were aware of the interviewers’ research interests. All 22 potential participants who expressed interest were eligible, one was lost to contact, leaving 21 who completed the interviews.

Survey participants were recruited via an external cross-panel market research provider (Octopus Group) 8 November 2021 to 21 November 2021. To be eligible, participants must have given birth within the past 5 years, not be pregnant, and live with the child in Australia. The study population was broadly representative of the Australian population according to the Australian Bureau of Statistics by location of residence (state or territory) [18].

### 2.3. Data Collection

The interview guide (Supplementary Materials File S1) was developed by female research dietitians experienced in developing lifestyle interventions, who also conducted the interviews (S.L. and L.J.M.). The interview guide was pilot tested on two postpartum women (not included in analyses) before the study. Semi-structured interviews were 30–40 min in duration and conducted 1:1 online via Zoom (Version: 5.4.2, Zoom Video Communications, San Jose, CA, USA, 2020) to facilitate accessibility to all participants. Interviews were audio-recorded and transcribed verbatim by a professional transcription service. Data saturation was achieved within the sample [19]. All interview participants were provided with the interview transcripts (member checking), of which four (20%) provided feedback and verification with no changes suggested.

The survey (Supplementary Materials File S2) was developed by the research team using the Qualtrics software. It was a self-administered 20–30 min online survey with questions on demographic characteristics; self-evaluation of capability, motivation, and opportunity for participation in lifestyle management adapted from the Capability, Opportunity, Motivation, Behaviour model, a behaviour change model for identifying what needs to change for interventions to be effective [20]; preferred intervention characteristics by TIDieR checklist [14]; psychological wellbeing [21]; co-parenting [22]; physical activity and sedentary time [23]; dietary intakes [24]; sleep patterns [25]; and risk perception for cardiovascular disease [26] and type 2 diabetes [27,28]. The current study only included analysis of the preferred intervention characteristics with questions based on the TIDieR checklist. The survey was pilot tested on four women (data not included in the analysis) and revised before the commencement of data collection.

### 2.4. Data Analysis

Qualitative data were coded and analysed using NVivo version 12 (QSR International Pty Ltd., Hawthorn East, VIC, Australia, 1999–2018). All interviews were coded by one researcher, and another independently coded a subsample (*n* = 2 for ~10% overlap). The coding was similar between the coders. The themes and subthemes emerging from the interviews were mapped to the TIDieR checklist and direct quotes from the transcript were used to illustrate each subtheme.

Descriptive statistics were used to summarize participant and program characteristics from quantitative data. Categorical data were reported as frequencies and percentages, while continuous data were reported as means and standard deviations for normally distributed variables or medians and interquartile ranges for non-normally distributed variables. Differences in participant and program characteristics by postpartum age (<2 years and 2–5 years) were explored using the *t*-test, Wilcoxon rank sum tests, and chi-square test as appropriate from two-tailed tests of statistical significance with a type 1 error rate of 5%. Stata software version 16 (Stata Corp, College Station, TX, USA) was used for analysis.

## 3. Results

### 3.1. Semi-Structured Interviews

The demographic characteristics of the participants (*n* = 21) are shown in Table 1. The interview participants were highly educated, and most were in paid employment. Four main themes emerged from the interviews namely, (1) practical strategies involving social support; (2) flexible and embedded routine care delivered by health professionals; (3) early and regular postpartum support; (4) manageable duration tailored to individual needs. Table 2 shows how each theme and subtheme maps on to the TIDieR checklist with direct quotes from participants to illustrate this.

#### 3.1.1. Theme 1: Practical Strategies Involving Social Support

Women consistently expressed the need for the practicality of intervention programs. They were interested in a program that provided practical support to deal with the challenges of the postpartum period and were not content with just receiving didactic information on healthy lifestyle behaviours but expressed concerns about information overload. Women considered practical support from their partners, family, and peers for childcare, very important to enable them to have some time for self-care. They also expressed the need for some form of accountability, someone to check on them and monitor their progress. Women preferred activities that could be done with children so that they would not have to worry about spending time away from their children or planning for childcare. Some mothers considered peer coaching as a useful component and a good source of support as long as the peer coaches had some qualifications. Others felt that some women have personal opinions about what a healthy lifestyle was and may try to impose those opinions on others during coaching.

#### 3.1.2. Theme 2: Flexible and Embedded Routine Care Delivered by Health Professional

Women wanted a program embedded within the routine services they were already engaging with and delivered by a health professional. They particularly favoured the maternal and child health (MCH) services (a free universal primary health service available to all families with children from birth to school age in Australia) [29] for program delivery and as a reliable source of health information. Participants also suggested health professionals such as general practitioners (GPs), physiotherapists, personal trainers, physiologists, psychologists, and dietitians as program providers. Having flexibility such as drop-in sessions which did not require making appointments was important to mothers because of the unpredictability of babies’ nap time. Women wanted in-person sessions to allow for effective communication, but also found online accessibility important. Both small group sessions and one-on-one sessions were acceptable to participants. In terms of cost, participants preferred a free service because on maternity leave, they are already on a reduced income. However, some expressed willingness to pay a small amount depending on who was rendering the service, e.g., they may be willing to pay for a health professional’s expertise.

#### 3.1.3. Theme 3: Early and Regular Postpartum Support

Women expressed a desire for early and regular postpartum lifestyle support. They acknowledged the challenge of starting too early because mothers need time to adjust to the newborn and find a regular sleep and feeding schedule. They however emphasized the need to start as early as possible before returning to work. Some women expressed a desire to start early because they get bored just staying home with the baby. In terms of program frequency, participants also wanted regular fortnightly or monthly sessions. Some stay-at-home mothers wanted weekly sessions to have something to go to more regularly.

#### 3.1.4. Theme 4: Manageable Duration Tailored to Individual Needs

In terms of session duration, women wanted manageable duration tailored to individual needs. Participants wanted short sessions of about 30 min and no more than 1 h, highlighting the difficulty of getting extended uninterrupted time with young children. They suggested that program intensity may vary depending on how well supported a mother feels and that program duration may be personalised to individual needs.

### 3.2. Survey

**Figure 1:**
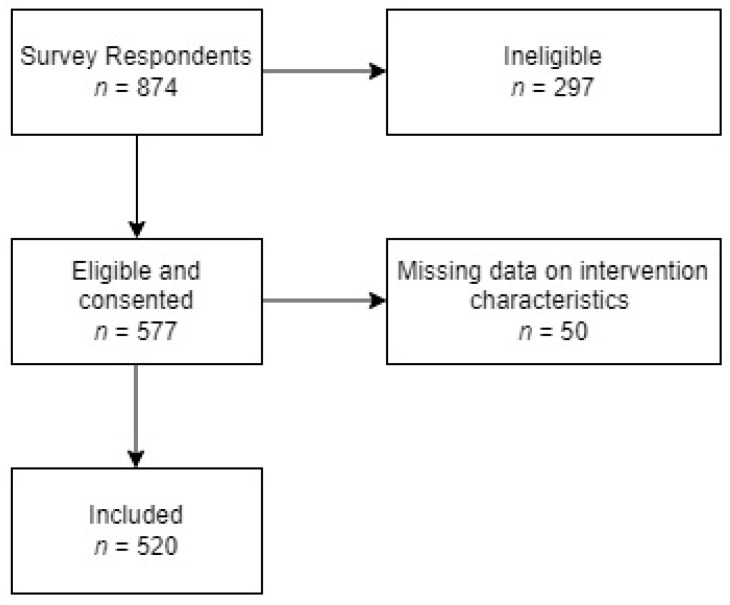
Flow chart of included survey participants.

There were 874 respondents to the survey of whom 577 were eligible and consented to participate. We further excluded 57 participants who were missing data on all intervention characteristics, leaving 520 participants included in the current study (Figure 1). Table 3 shows the demographic characteristics of survey participants. Most survey participants had a degree or more, were employed or studying, and were of medium income households.

Table 4 provides the survey responses for preferred intervention characteristics according to the TIDieR checklist. Most of the participants (90.4%) were interested in receiving postpartum lifestyle support. The preferred program contents were information on women’s health (83.3%), mental health (76.2%), and exercise after birth (71.0%). They also wanted information on a range of health issues including breastfeeding, children’s health, diet, infant care, and weight issues. More than 60% of respondents preferred a program that included social support for health and someone to monitor their progress. Women with children <2 years were more likely to desire information on exercise after birth than women with children 2–5 years (81.4% versus 63.4%) and on how to determine the credibility of health information (32.7% versus 24.2%). The most preferred program provider were health professionals with expertise in women’s health (90%). More women with children <2 years (30.8%) than those with children 2–5 years (22.5%) wanted program delivery by another mum. The most preferred delivery mode was online information and resource (74%), but more than half also wanted individual face-to-face consultation (55.2%). The most preferred delivery platform was through MCH nurse visits (75%). The most preferred avenue to learn about an intervention program was through a health facility (85%) followed by social media (77.3%). Almost half of the women with children <2 years also wanted to learn about the program through playgroups, mothers, or parents groups. A larger proportion of women wanted an early start of 7 weeks–3 months (40.4%) and short session durations of 15–30 min (43.5%).

## 4. Discussion

This is the first study to explore the preferences of women for a postpartum lifestyle intervention program using the TIDieR template to facilitate a description of the intervention components and context. Both surveys and interviews revealed that women wanted to receive support for healthy lifestyles in the postpartum period. Overall, 19–20% of participants had gestational diabetes during their pregnancy, which is slightly above the incidence rate of 17% at population level [30]. Women were interested in programs that were practical, supportive, flexible, integrated into routine care, and delivered by health professionals. They wanted regular support that started in the early postpartum period with a manageable duration that is tailored to individual needs. Women with children <2 years were more likely to be interested in learning about exercise after birth and the credibility of health information compared to those with children 2–5 years. They were also more likely to prefer delivery of intervention by another mum and to want to learn about lifestyle programs through playgroups, mothers or parents groups or a health facility than other avenues.

This study highlights the mismatch between postpartum women’s preferences for lifestyle support and current practice. From the preferences that women discussed it seems that there is a need for postpartum lifestyle interventions to shift from focusing on the provision of didactic information to teaching practical strategies to mitigate barriers to healthy lifestyle behaviours [31]. Such strategies could include meal planning and preparation ideas, how to use seasonal fruits and vegetables to minimise cost, cooking healthy on a budget, time management, and motivational tools such as reminders, self-monitoring, and reinforcement [13,32,33]. Addressing the personal and environmental mediators of behaviour rather than delivering factual information is most likely to lead to the desired outcome [34]. Survey participants wanted the inclusion of information on a range of health issues specifically around women’s health, mental health and exercise after childbirth suggesting that information provision is still an important component of postpartum lifestyle interventions. Of note though, is that interview participants were very highly educated and felt they could easily access any information they wanted online. Survey participants however, especially those with children <2 years were interested in knowing how to determine the credibility of health information. Therefore, interventions may need to be tailored differently depending on the educational levels of the target population.

Both interview and survey findings suggest that social support for childcare, cooking, and physical activity is an important component of lifestyle programs for postpartum women [31]. Social support is very important for healthy eating and engaging in regular physical activity in all population groups and especially for postpartum women because of the challenges of caring for an infant [13,31,35]. Previous studies have shown that mothers that feel well supported were more likely to engage in healthy lifestyle behaviours than those who did not feel supported [31,35]. Enlisting the support of partners and family members through a family focused intervention approach may be an important addition to postpartum lifestyle programs [13,31,36].

Peer support is also an important source of support, especially for mothers who live far from other family members. In our study, having mothers groups as a source of peer support especially when members were of similar cultural backgrounds was important to interview participants. Peer support groups facilitated by the MCH nurse have been reported to increase the confidence of participating mothers around parenting and infant care and provide social connections [37,38]. Although peer coaching was considered a relevant program addition by some mothers, especially when the coaches had some relevant qualifications, others viewed it as irrelevant because of the potential for mothers to impose their personal opinions on others. Therefore, care should be taken when including peer coaching in a program to ensure that mothers are matched with coaches of similar backgrounds or cultures, values, and lived experiences to build trust and credibility [39]. Ensuring that peer coaches are adequately trained may also help mitigate these concerns.

The involvement of health professionals has previously been reported as an important component of successful lifestyle interventions for postpartum women [40]. In both interviews and surveys, participants indicated a preference for intervention delivery by health professionals, particularly favouring the MCH service. In Australia, the MCH service is a universal primary healthcare service to promote child health and development and provide parental support [29]. Interventions integrated within routine MCH visits were shown to be effective in reducing postpartum weight retention in previous studies [41,42]. This may be because the barriers faced by postpartum women such as time constraints and the need for childcare are minimised when interventions are integrated into the usual schedules of postpartum women [13,42]. Research suggests that health professionals are willing to provide support for postpartum women, but are often limited by time constraints and limited skills [13]. There may therefore be need for a systems approach to integrating postpartum lifestyle interventions into existing health services such as the MCH service.

Flexibility of programs was considered important by interview participants. This can be achieved either through embedding in routine services as discussed above or providing online accessibility. Increasing the accessibility of intervention programs increases the proportion of the target population reached by the intervention leading to greater population impact [43]. An online mode of delivery which was also acceptable to survey participants may alleviate some of the barriers to engagement although, face-to-face delivery may be beneficial for effective communication. Previous studies combining both face-to-face and online delivery modes have demonstrated effectiveness for weight loss during the postpartum period [42,44]. A recent survey study reported that most postpartum women preferred a combination of online and face-to-face sessions for psychological intervention for postpartum depression [45].

In our interview study, women suggested that group sessions facilitated social engagement and learning from the experiences of others while one-on-one sessions were beneficial for counselling and personalised support. A previous study reported a significant increase in engagement of postpartum women in an intervention program when the delivery mode was changed from group-based face-to-face to telephone-based one-on-one sessions [46]. In that study, the increase in engagement was attributed to alleviating the barriers to access. Therefore, accessibility of intervention programs irrespective of the delivery mode is most important to achieve engagement leading to large-scale impact. Therefore, having combinations of individual and group sessions in face-to-face and online settings may be most suited to the needs of postpartum mothers.

Interview and survey participants had variable preferences on intervention commencement time after birth, frequency and session duration. This is consistent with evidence from a systematic review that suggests that intervention duration and the number of sessions are adaptable elements of an intervention [40]. Therefore, individual circumstances and contextual factors should be considered when deciding on these elements of intervention for postpartum women. For example, in our interviews, stay-at-home mums preferred a more intensive program than working mums, suggesting that interventions may need to be tailored differently for working mothers compared to stay-at-home mothers.

### Strengths and Limitations

A strength of this study is the multi-methods approach. Employing complementary qualitative and quantitative methods enriched our understanding of the preferences of women for a lifestyle intervention in the postpartum period. We were able to combine the scientific objectivity and generalizability afforded by quantitative methods with a rich understanding of context that can only be obtained through qualitative interviews with postpartum women. Secondly, the qualitative part of this study was conducted and reported according to the COREQ guidelines to enhance data credibility, dependability, and confirmability. Thirdly, having a large population (>40%) of overseas-born participants and representativeness across all Australian states increases the transferability of the findings across Australian populations. Fourthly, having a large number of participants in the survey, and data saturation in the qualitative interviews indicates the robustness of the results [47]. Lastly, understanding the perspectives of program end users helps to ensure that programs are consumer-centred and acceptable.

## 5. Conclusions

Postpartum women have unique preferences regarding the delivery of lifestyle intervention programs, and understanding what they are is important for the development of effective lifestyle interventions. Capturing the preferences of postpartum women for a lifestyle intervention program will ensure that interventions are consumer-informed, thereby increasing program acceptability and engagement. Practical interventions that leverage existing universal health services and are embedded within routine care are acceptable to postpartum women and amenable for implementation at scale in real-world settings. Our findings are relevant to inform researchers and policymakers engaged in the development of postpartum lifestyle interventions on approaches that ensure effective implementation and scale-up.

## Figures and Tables

**Table 1:** Characteristics of interview participants (*n* = 21).

Characteristics	Interviews *n* (%) ^1^
Age (mean ± SD)	36.5 ± 4.3
BMI (median ± IQR)	23.1 ± 3.2
Age of youngest child	
Less than 6 months	4 (19.0)
6 months to less than 1 year	1 (4.8)
1 year old	16 (76.2)
Country of birth	
Australian born	13 (61.9)
Overseas born	8 (38.1)
Education	
High school	1 (4.8)
Diploma/Advanced diploma	0
Degree/higher	20 (95.2)
Employment	
Unemployed/homemaker	2 (9.5)
Employed/studying	19 (90.5)
Complication in pregnancy	
Gestational diabetes	4 (19.0)
Preeclampsia	1 (4.8)

SD, standard deviation; BMI, body mass index; IQR, interquartile range; ^1^ Frequencies (*n*) and percentages (%) reported unless stated otherwise.

**Table 2:** Intervention characteristics according to the TIDieR checklist from the perspective of postpartum women (*n* = 21).

TIDieR Element	Subtheme	Theme	Representative Quotes ^1^
What (program content or type)	No need for more information	Theme 1: Practical strategies involving social support	Uh, to be honest, I don’t really feel like I need more information. I feel like (laughs) at the moment it’s information overload (laughs). #3, 43
	Involve partners, family and mothers groups as the main sources of support		It’s helpful if, um, if we find some mums that really, um, similar- similar- similar background for myself, yeah. #1, 44
	Practical support and activities that can be done with children		So any, any sort of service that can help with allowing you to have more time, um, I think would be great. #7, 34
	Practical strategies and accountability		…whatever the model is, it’s about having, you know, accountability to keep you on that track…. I just think something where you’ve got people who check in on you who make that, who keep you consistent. #19, 42
	Peer coaching		Yeah, I think that is a good source of support because we understand what each other is going through and what some of the barriers may be to looking after our health. Getting out and about with other mothers and exercising is really great. #6, 39
Who (program provider)	Maternal and child health nurses	Theme 2: Flexible and embedded routine care delivered by health professional	in those early days given the amount of, um, interaction you have with the maternal and child health nurse- that would probably be a really good avenue because certainly they’re already talking to you around, you know, they’re doing the depression screener and things. #12, 37
	Other health professionals		And for me a GP not so much, because I don’t really have any major health problems. But Allied Health, a personal trainer exercise, you know, a physiologist who could set a program would be good. #13, 33
How (delivery mode and setting)	Embedded within regular schedules of infant care		if it’s something that fits into your regular routine, so if you’re seeing those mothers on a regular basis and it’s something that you could implement #19, 42
	Small groups or one-on-one		One-on-one might be… group is always nice, because you hear other people’s experiences, too, and sometimes then you can open up and share, but one-on-one with the mat nurse is, is okay, too. Either, I think either would be okay. #11, 31
	Flexibility		…having sessions …that set up or even the option to drop in and have those discussions as you need. Um, I think that’s really important because I know that with a young child at home, um, having appointments or having set times and days for things isn’t always possible… #9, 30
Where (delivery platform)	Child-friendly space		I’m kind of imagining like a bit of a creche area or something like that. Where your… Where the babies have like a safe area to be. Maybe someone who can kind of watch them, but you’re still right there, like you can see them and um, yeah. #21, 34
	Online accessibility		I would choose online. group environment. #14, 38
	Face-to-face		Um, I think, I think, it’s better to meet personally than online just- just only… I mean, online probably for information but, yeah, we still need that social interaction. #1, 44
	Maternal and child health centre		Yeah, I mean I guess um, the maternal child health nurse would be the obvious thing, because that’s, I was going there anyway you know, fairly regularly. So we could have had that incorporated into something I’m already doing. #3, 43
Program cost	Free or small cost		…having something provided for you that is free of charge I think is, um, I, yeah. I just think it’s, um, it’s a good way to make you feel supported. #4, 38
When and how much (program commencement)	3–6 months after birth	Theme 3: Early and regular postpartum support	…after six months probably….Cause the first, the early month is, um, it’s just tiring and busy with, uh, feeding and you know, adjusting. #2, 43
When and how much (How often—program frequency)	Weekly		So yeah. Look, when you’re at home and you don’t have much to do or go to, I really liked having something to go to every week. #5, 32
	Fortnightly or monthly		But now like with work, I think once, uh, once fortnightly or something like that will be nice to have like a group where you can go on talk or discussions or even to activities or go out for something, whatever. #15, 38
When and how much (length of session)	Short sessions of about 30 min and not more than 1 h	Theme 4: Manageable duration tailored to individual needs	Yeah, yeah, up to an hour. An hour’s a long time for a baby. #21, 34
	Depends on the nature of the support		I’m quite happy to receive the information and then do the work on my own but other women may need or want more support. … but I’m sure there’s plenty of mums out there that would actually like more frequent intensive support. It’s a personal choice depending on how well supported you may feel. #6, 39
When and how much (program duration)	As needed		yeah probably, as regular as you need them there after …I think as long as, if they worked… in tandem with those, um, maternity health care appointments. That could work. #16, 33
	Short term		I think long term, people lose interest. But this is me being biased. #13, 33

^1^ (# Assigned number, age of participant).

**Table 3:** Characteristics of survey participants (*n* = 520).

**Characteristics**	**Whole Population** **(*n* = 520)** ***n*** **(%) ^1^**	**Women with Children < 2 Years (*n* = 214) *n* (%)**	**Women with Children 2–5 Years (*n* = 306)** ***n*** **(%)**	***p*** **Value ^2^**
Age (mean ± SD)	33.6 ± 5.4	31.7 ± 5.0	35.0 ± 5.3	<0.001
BMI (median ± IQR)	25.7 ± 9.4	25.7 ± 9.4	25.6 ± 9.2	0.9201
Age of youngest child				<0.001
Less than 6 months	62 (11.9)	62 (29.0)	N/A	
6 months to less than 1 year	66 (12.7)	66 (30.8)	N/A	
1 year old	86 (16.5)	86 (40.2)	N/A	
2 years old	95 (18.3)	N/A	95 (31.1)	
3 years old	73 (14.0)	N/A	73 (23.9)	
4 years old	70 (13.5)	N/A	70 (22.9)	
5 years old	68 (13.1)	N/A	68 (22.2)	
Country of birth				0.833
Australian born	282 (54.2)	125 (58.4)	157 (51.3)	
Overseas born	238 (45.8)	89 (41.6)	149 (48.7)	
Marital status				0.005
Married/de facto	450 (88.5)	194 (90.7)	266 (86.9)	
Single (never married/divorced/separated)	57 (11.0)	18 (8.4)	39 (12.8)	
Missing	3 (0.6)	2 (0.9)	1 (0.3)	
Education				0.751
High school	128 (24.6)	54 (25.2)	74 (24.2)	
Diploma/Advanced diploma	101 (19.4)	36 (16.8)	65 (21.2)	
Degree/higher	288 (55.4)	124 (57.9)	164 (53.6)	
Missing	3 (0.6)	0	3 (1.0)	
Employment				0.535
Unemployed/homemaker	147 (28.3)	65 (30.4)	82 (26.8)	
Employed/studying	365 (70.2)	145 (67.8)	220 (71.9)	
Missing	8 (1.5)	4 (1.9)	4 (1.3)	
Annual Household Income				0.192
Low (<50,000 AUD)	80 (15.4)	24 (11.2)	56 (18.3)	
Medium (50,000–124,999 AUD)	247 (47.5)	107 (50.0)	140 (45.8)	
High (≥125,000)	161 (31.0)	72 (33.6)	89 (29.1)	
Missing	32 (6.2)	11 (5.1)	21 (6.9)	
Pregnancy complications				
Gestational diabetes	105 (20.2)	50 (23.4)	55 (18.0)	0.149
Preeclampsia	35 (6.7)	15 (7.0)	20 (6.5)	0.860
Gestational hypertension	38 (7.3)	20 (9.4)	18 (5.9)	0.170
Small for gestational age	23 (4.4)	13 (6.1)	10 (3.3)	0.135
Pre-term birth	64 (12.3)	29 (13.6)	35 (11.4)	0.499
Medical conditions				
Diabetes	17 (3.3)	5 (2.3)	12 (3.9)	0.453
Polycystic ovary syndrome	45 (8.7)	12 (5.6)	33 (10.8)	0.040
Infertility	29 (5.6)	8 (3.7)	21 (6.9)	0.173

SD, standard deviation; BMI, body mass index; IQR, interquartile range; N/A, not applicable; ^1^ Frequencies (*n*) and percentages (%) reported unless where stated otherwise; ^2^ Differences in participant characteristics between women with children <2 years and 2–5 years were explored using *t*-test, Wilcoxon rank sum tests, and chi-square test as appropriate with significance level of 0.05.

**Table 4:** Survey responses for preferred intervention characteristics according to the TIDieR checklist (*n* = 520).

TIDieR Element	Program Characteristics	Responses, *n* (%) (Whole Population, *n* = 520)	Responses, *n* (%) (Women with Children <2 years, *n* = 214)	Responses, *n* (%) (Women with Children 2–5 years, *n* = 306)	*p* Value ^1^
Interest in a lifestyle program	Yes	470 (90.4)	196 (91.6)	274 (89.5)	0.455
	No	50 (9.6)	18 (8.4)	32 (10.5)	
What (program content) (multiple response question)	Women’s health	433 (83.3)	175 (81.8)	258 (84.3)	0.475
	Breastfeeding	351 (67.5)	152 (71.0)	199 (65.0)	0.155
	Caring for my baby	326 (62.7)	137 (64.0)	189 (61.8)	0.645
	Children’s health	343 (66.0)	141 (65.9)	202 (66.0)	1.000
	Mother’s diet	336 (64.6)	140 (65.4)	196 (64.1)	0.780
	How to lose weight	306 (58.8)	117 (54.7)	189 (61.8)	0.124
	How to prevent weight gain	243 (46.7)	94 (43.9)	149 (48.7)	0.286
	How to maintain weight	226 (43.5)	88 (41.1)	138 (45.1)	0.419
	Preventing diabetes or heart disease	169 (32.5)	69 (32.2)	100 (32.7)	0.925
	Mental health	396 (76.2)	168 (78.5)	228 (74.5)	0.298
	Exercise after birth	369 (71.0)	175 (81.8)	194 (63.4)	<0.001
	How to determine the credibility of health information	144 (27.2)	70 (32.7)	74 (24.2)	0.037
	How to set goals and action plans for health	236 (45.4)	98 (45.8)	138 (45.1)	0.929
	How to set aside time for health	259 (49.8)	115 (53.7)	144 (47.1)	0.154
	Self-recording diet and physical activity	145 (27.9)	61 (28.5)	84 (27.5)	0.843
	Monitoring blood tests and other health outcomes	130 (25.0)	55 (25.7)	75 (24.5)	0.759
	Others—e.g., body dysmorphia after birth, focusing on becoming fitter and stronger rather than losing weight, how to manage postnatal depression, learning about eczema, relationship, and sex after children, pelvic floor rehabilitation	7 (1.3)	6 (2.8)	1 (0.3)	0.021
What (additional program inclusions) (multiple response question)	Someone to monitor my progress	326 (62.7)	137 (64.0)	189 (61.8)	0.645
	Send me reminders and prompts	299 (57.5)	127 (59.3)	172 (56.2)	0.476
	Social support for health	337 (64.8)	146 (68.2)	191 (62.4)	0.192
	Questions to ask my doctor	216 (41.5)	98 (45.8)	118 (38.6)	0.104
	Others, e.g., include something for the child’s father, physio, mental health is vital postpartum, like someone to check in, but respect wishes and not insist	5 (1.0)	1 (0.5)	4 (1.3)	0.653
Who (program provider) (multiple response question)	Someone with expertise in women’s health, e.g., health professional	468 (90.0)	194 (90.7)	274 (89.5)	0.678
	Someone with expertise in children’s health, e.g., health professional	283 (54.4)	114 (53.3)	169 (55.2)	0.659
	Another mum	135 (26.0)	66 (30.8)	69 (22.5)	0.042
	Someone else—Dietitian, GP, psychologist, postpartum midwife, registered nurse, personal trainer, any person other than a health professional, holistic person, young non-menopausal woman, maternal and child health nurse, someone knowledgeable in nutrition and women’s recovery after childbirth	10 (1.9)	5 (2.3)	5 (1.6)	0.747
How (delivery mode and setting) (multiple response question)	Online information and resource	385 (74.0)	165 (77.1)	220 (71.9)	0.188
	Print information and resource	191 (36.7)	70 (32.7)	121 (39.5)	0.117
	One-on-one video or phone consultation	214 (41.2)	90 (42.1)	124 (40.5)	0.786
	One-on-one face-to-face consultation	287 (55.2)	120 (56.1)	167 (54.6)	0.788
	Group video consultation	119 (22.9)	44 (20.6)	75 (24.5)	0.340
	Group face-to-face consultation	194 (37.3)	84 (39.3)	110 (35.9)	0.462
	Others—nurse home visits	1 (0.2)	1 (0.5)	0	N/A
Where (delivery platform) (multiple response question)	Online	352 (67.7)	146 (68.2)	206 (67.3)	0.849
	Maternal child health nurse visit	390 (75.0)	164 (76.6)	226 (73.9)	0.537
	Mothers group/playgroup	279 (53.7)	116 (54.2)	163 (53.3)	0.858
	GP clinic	269 (51.7)	114 (53.3)	155 (50.7)	0.593
	Others—at a paediatric appointment, centre, home visit, in-home or at a gym, maternity ward, in own home, women’s health professional	1 (0.2)	4 (1.9)	3 (1.0)	0.454
Where (avenue for learning about the program)	Social media (Facebook, Twitter, Instagram, WhatsApp, WeChat, LINE)	402 (77.3)	163 (76.2)	239 (78.1)	0.604
	Word of mouth	222 (42.7)	92 (43.0)	130 (42.5)	0.928
	Blog or Forum, e.g., Blog, Google, Healthengine, Mum	21 (4.0)	9 (4.2)	12 (3.9)	1.000
	Newspapers, e.g., Herald sun, 7news, 9news, ABC news, online newspapers	15 (2.9)	6 (2.8)	9 (2.9)	1.000
	Playgroup/Mothers group/Parents group	230 (44.2)	106 (49.5)	124 (40.5)	0.048
	School, childcare, or early learning centre	199 (38.3)	77 (36.0)	122 (39.9)	0.409
	Public library	103 (19.8)	42 (19.6)	61 (19.9)	1.000
	Health facility (hospital, GP clinic, MCH nurse or centre)	442 (85.0)	193 (90.2)	249 (81.4)	0.006
	Others, e.g., email, Google search, letters sent directly, survey	5 (1.0)	1 (0.5)	4 (1.3)	
When and how much (program commencement)	6 weeks or earlier	171 (32.9)	64 (29.9)	107 (35.0)	0.306
	7 weeks to 3 months	210 (40.4)	90 (42.1)	120 (39.2)	
	4—6 months	98 (18.9)	44 (20.6)	54 (17.6)	
	7–12 months	18 (3.5)	5 (2.3)	13 (4.2)	
	After 12 months	7 (1.4)	2 (0.9)	5 (1.6)	
	Other—preconception, during pregnancy, straight away, between 0 and 8 weeks at earliest but it can be overwhelming, every woman is different, as soon as possible because it feels like the mother’s wellbeing is forgotten about too quickly after it feels right—it can be 1 month or 12 months	9 (1.7)	6 (2.8)	3 (1.0)	
	Missing	7 (1.4)	3 (1.4)	4 (1.3)	
When and how much (how often—program frequency)	Every 6 months	27 (5.2)	7 (3.3)	20 (6.6)	0.346
	Every 3 months	94 (18.1)	36 (16.8)	58 (19.2)	
	Every month	190 (36.5)	84 (39.3)	106 (35.1)	
	Every fortnight	121 (23.3)	49 (22.9)	72 (23.8)	
	Every week	70 (13.5)	29 (13.6)	41 (13.6)	
	Once off	4 (0.8)	1 (0.5)	3 (1.0)	
	Others—every day, every 3 weeks, valuable in an app—on demand, every month for 4 months and then quarterly thereafter, an individual plan that caters to the mum and her family	7 (1.4)	5 (2.3)	2 (0.7)	
	Missing	7 (1.4)	3 (1.4)		
When and how much (length of session)	Less than 15 min	30 (5.8)	12 (5.6)	18 (6.0)	0.891
	Between 15 and 30 min	226 (43.5)	94 (43.9)	132 (43.7)	
	Between 30 and 45 min	181 (34.8)	74 (34.6)	107 (35.4)	
	Between 45 and 60 min	71 (13.7)	28 (13.1)	43 (14.2)	
	More than 60 min	4 (0.8)	2 (0.9)	2 (0.7)	
	Others	1 (0.2	1 (0.5)	0	
	Missing	7 (1.4)	3 (1.4)	4 (1.3)	
When and how much (program duration)	<1 month	19 (3.7)	6 (2.8)	13 (4.3)	0.899
	1 month	47 (9.0)	19 (8.9)	28 (9.3)	
	3 months	73 (14.0)	30 (14.0)	43 (14.2)	
	6 months	133 (25.6)	59 (27.6)	74 (24.5)	
	1 year	235 (45.2)	94 (43.9)	141 (46.7)	
	Others—2 years, as long as it takes to lose the weight, as long as needed, however long or short you want, long term	6 (1.2)	3 (1.4)	3 (1.0)	
	Missing	7 (1.4)	3 (1.4)	4 (1.3)	

^1^ Differences in participant characteristics between women with the youngest child <2 years and 2–5 years were explored using *t*-test, Wilcoxon rank sum tests, and chi-square test as appropriate with significance level of 0.05. GP, general practitioner.

## Data Availability

The data presented in this study are available on request from the corresponding author. The data are not publicly available because it is potentially identifiable.

## Supplementary Materials

The following supporting information can be downloaded at: https://www.mdpi.com/article/10.3390/nu14204229/s1, Supplementary File S1: Interview guide; Supplementary File S2: Online survey.

## References

[1] Endres L.K., Straub H., McKinney C., Plunkett B., Minkovitz C.S., Schetter C.D., Ramey S., Wang C., Hobel C., Raju T. (2015). Postpartum weight retention risk factors and relationship to obesity at one year. Obstet. Gynecol..

[2] McKinley M.C., Allen-Walker V., McGirr C., Rooney C., Woodside J.V. (2018). Weight loss after pregnancy: Challenges and opportunities. Nutr. Res. Rev..

[3] Abebe D.S., Von Soest T., Von Holle A., Zerwas S.C., Torgersen L., Bulik C.M. (2015). Developmental trajectories of postpartum weight 3 years after birth: Norwegian Mother And Child Cohort study. Matern. Child Health J..

[4] Mannan M., Doi S.A., Mamun A.A. (2013). Association between weight gain during pregnancy and postpartum weight retention and obesity: A bias-adjusted meta-analysis. Nutr. Rev..

[5] Nehring I., Schmoll S., Beyerlein A., Hauner H., von Kries R. (2011). Gestational weight gain and long-term postpartum weight retention: A meta-analysis. Am. J. Clin. Nutr..

[6] Makama M., Skouteris H., Moran L.J., Lim S. (2021). Reducing Postpartum Weight Retention: A Review of the Implementation Challenges of Postpartum Lifestyle Interventions. J. Clin. Med..

[7] Gore S.A., Brown D.M., West D.S. (2003). The role of postpartum weight retention in obesity among women: A review of the evidence. Ann. Behav. Med..

[8] Kew S., Ye C., Hanley A.J., Connelly P.W., Sermer M., Zinman B., Retnakaran R. (2014). Cardiometabolic implications of postpartum weight changes in the first year after delivery. Diabetes Care.

[9] Farpour-Lambert N.J., Ells L.J., Martinez de Tejada B., Scott C. (2018). Obesity and weight gain in pregnancy and postpartum: An evidence review of lifestyle interventions to inform maternal and child health policies. Front. Endocrinol..

[10] Lim S., Hill B., Teede H.J., Moran L.J., O’Reilly S. (2020). An evaluation of the impact of lifestyle interventions on body weight in postpartum women: A systematic review and meta-analysis. Obes. Rev..

[11] Lim S., O’Reilly S., Behrens H., Skinner T., Ellis I., Dunbar J. (2015). Effective strategies for weight loss in post-partum women: A systematic review and meta-analysis. Obes. Rev..

[12] Jones E.J., Fraley H.E., Mazzawi J. (2017). Appreciating recent motherhood and culture: A systematic review of multimodal postpartum lifestyle interventions to reduce diabetes risk in women with prior gestational diabetes. Matern. Child Health J..

[13] Makama M., Awoke M.A., Skouteris H., Moran L.J., Lim S. (2021). Barriers and facilitators to a healthy lifestyle in postpartum women: A systematic review of qualitative and quantitative studies in postpartum women and healthcare providers. Obes Rev..

[14] Hoffmann T.C., Glasziou P.P., Boutron I., Milne R., Perera R., Moher D., Altman D.G., Barbour V., Macdonald H., Johnston M. (2014). Better reporting of interventions: Template for intervention description and replication (TIDieR) checklist and guide. Bmj.

[15] Cotterill S., Knowles S., Martindale A.-M., Elvey R., Howard S., Coupe N., Wilson P., Spence M. (2018). Getting messier with TIDieR: Embracing context and complexity in intervention reporting. BMC Med. Res. Methodol..

[16] Anguera M.T., Blanco-Villaseñor A., Losada J.L., Sánchez-Algarra P., Onwuegbuzie A.J. (2018). Revisiting the difference between mixed methods and multimethods: Is it all in the name?. Qual. Quant..

[17] Tong A., Sainsbury P., Craig J. (2007). Consolidated criteria for reporting qualitative research (COREQ): A 32-item checklist for interviews and focus groups. Int. J. Qual. Health Care.

[18] Australian Bureau of Statistics (2021). National, State and Territory Population. https://www.abs.gov.au/statistics/people/population/national-state-and-territory-population/latest-release#.

[19] Guest G., Bunce A., Johnson L. (2006). How many interviews are enough? An experiment with data saturation and variability. Field Methods.

[20] Michie S., Van Stralen M.M., West R. (2011). The behaviour change wheel: A new method for characterising and designing behaviour change interventions. Implement. Sci..

[21] Kessler R.C., Andrews G., Colpe L.J., Hiripi E., Mroczek D.K., Normand S.-L., Walters E.E., Zaslavsky A.M. (2002). Short screening scales to monitor population prevalences and trends in non-specific psychological distress. Psychol Med..

[22] Feinberg M.E., Brown L.D., Kan M.L. (2012). A Multi-Domain Self-Report Measure of Coparenting. Parent Sci. Pract..

[23] Brown W.J., Burton N.W., Marshall A.L., Miller Y.D. (2008). Reliability and validity of a modified self-administered version of the Active Australia physical activity survey in a sample of mid-age women. Aust. N. Z. J. Public Health.

[24] Malek L., Umberger W., Makrides M., Zhou S.J. (2016). Adherence to the Australian dietary guidelines during pregnancy: Evidence from a national study. Public Health Nutr..

[25] Buysse D.J., Yu L., Moul D.E., Germain A., Stover A., Dodds N.E., Johnston K.L., Shablesky-Cade M.A., Pilkonis P.A. (2010). Development and validation of patient-reported outcome measures for sleep disturbance and sleep-related impairments. Sleep.

[26] Traylor J., Chandrasekaran S., Limaye M., Srinivas S., Durnwald C.P. (2016). Risk perception of future cardiovascular disease in women diagnosed with a hypertensive disorder of pregnancy. J. Matern. Fetal Neonatal Med..

[27] Walker E.A., Mertz C.K., Kalten M.R., Flynn J. (2003). Risk perception for developing diabetes: Comparative risk judgments of physicians. Diabetes Care.

[28] Kim C., McEwen L.N., Piette J.D., Goewey J., Ferrara A., Walker E.A. (2007). Risk perception for diabetes among women with histories of gestational diabetes mellitus. Diabetes Care.

[29] Rossiter C., Fowler C., Hesson A., Kruske S., Homer C.S., Schmied V. (2019). Australian parents’ use of universal child and family health services: A consumer survey. Health Soc. Care Community.

[30] Australian Institute of Health and Welfare (2022). Diabetes: Australian facts.

[31] Ingstrup M.S., Wozniak L.A., Mathe N., Butalia S., Davenport M.H., Johnson J.A., Johnson S.T. (2019). Women’s experience with peer counselling and social support during a lifestyle intervention among women with a previous gestational diabetes pregnancy. Health Psychol. Behav. Med..

[32] MacKenzie-Shalders K., Matthews C., Dulla J., Orr R. (2020). Law enforcement personnel are willing to change, but report influencing beliefs and barriers to optimised dietary intake. BMC Public Health.

[33] Lim S., Hill B., Pirotta S., O’Reilly S., Moran L. (2020). What Are the Most Effective Behavioural Strategies in Changing Postpartum Women’s Physical Activity and Healthy Eating Behaviours? A Systematic Review and Meta-Analysis. J. Clin. Med..

[34] Contento I.R. (2008). Nutrition education: Linking research, theory, and practice. Asia Pac. J. Clin. Nutr..

[35] Carter-Edwards L., Østbye T., Bastian L.A., Yarnall K.S., Krause K.M. (2009). Barriers to adopting a healthy lifestyle: Insight from postpartum women. BMC Res. Notes.

[36] Nicklas J.M., Zera C.A., Seely E.W., Abdul-Rahim Z.S., Rudloff N.D., Levkoff S.E. (2011). Identifying postpartum intervention approaches to prevent type 2 diabetes in women with a history of gestational diabetes. BMC Pregnancy Childbirth.

[37] Kruske S., Schmied V., Sutton I., O’Hare J. (2004). Mothers’ experiences of facilitated peer support groups and individual child health nursing support: A comparative evaluation. J. Perinat Educ..

[38] Scott D., Brady S., Glynn P. (2001). New mother groups as a social network intervention: Consumer and maternal and child health nurse perspectives. Aust. J. Adv. Nurs..

[39] Herring S.J., Bersani V.M., Santoro C., McNeil S.J., Kilby L.M., Bailer B. (2021). Feasibility of using a peer coach to deliver a behavioral intervention for promoting postpartum weight loss in Black and Latina mothers. Transl. Behav. Med..

[40] Lim S., Liang X., Hill B., Teede H., Moran L.J., O’Reilly S. (2019). A systematic review and meta-analysis of intervention characteristics in postpartum weight management using the TIDieR framework: A summary of evidence to inform implementation. Obes. Rev..

[41] Harrison C.L., Lombard C.B., Teede H.J. (2014). Limiting postpartum weight retention through early antenatal intervention: The HeLP-her randomised controlled trial. Int. J. Behav. Nutr. Phys. Act..

[42] Huseinovic E., Bertz F., Leu Agelii M., Hellebö Johansson E., Winkvist A., Brekke H.K. (2016). Effectiveness of a weight loss intervention in postpartum women: Results from a randomized controlled trial in primary health care. Am. J. Clin. Nutr..

[43] Dasgupta K., Maindal H.T., Nielsen K.K., O’Reilly S. (2018). Achieving penetration and participation in diabetes after pregnancy prevention interventions following gestational diabetes: A health promotion challenge. Diabetes Res. Clin. Pract..

[44] Colleran H.L., Lovelady C.A. (2012). Use of MyPyramid Menu Planner for Moms in a weight-loss intervention during lactation. J. Acad. Nutr. Diet..

[45] Branquinho M., Canavarro M.C., Fonseca A. (2021). A blended psychological intervention for postpartum depression: Acceptability and preferences in women presenting depressive symptoms. J. Reprod. Infant Psychol..

[46] Lim S., Dunbar A.J., Versace V., Janus E., Wildey C., Skinner T., O’Reilly S. (2017). Comparing a telephone-and a group-delivered diabetes prevention program: Characteristics of engaged and non-engaged postpartum mothers with a history of gestational diabetes. Diabetes Res. Clin. Pract..

[47] Frambach J.M., van der Vleuten C.P., Durning S.J. (2013). AM last page. Quality criteria in qualitative and quantitative research. Acad. Med..

