# Supplementary material for: Postpartum Women’s Preferences for Lifestyle Intervention after Childbirth: A Multi-Methods Study Using the TIDieR Checklist
Source: Nutrients. 2022 Oct 11;14(20):4229. doi: 10.3390/nu14204229 (PMC9611337; doi:10.3390/nu14204229)
Supplement: Supplementary file 1 [file nutrients-14-04229-s001.zip › nutrients-1943186-supplementary/Supplementary file S1.pdf]

Supplementary file S1: Interview guide

A) Housekeeping:

Thank her for her time. Obtain consent to participate in the interview and to be recorded

B) Orientation questions

Ice-breakers<br>How long have you lived here?<br>Who else do you live with?<br>How many children to you have? How old are they?
CALD background (if relevant)<br>Were you born in Australia? How long since you've moved to Australia? Did you speak English before?

C) Perspectives and practice on healthy lifestyles

Perspectives<br>We are studying about healthy lifestyle but that means different things to different people. What do you think a healthy lifestyle is?<br><br>• Diet<br>• Exercise<br>• Mental health<br>• Sleep<br>• Other<br>• Other aspects – eg stress management, sleep
Lifestyle and health consequences: Mum<br>What is your greatest health concern now?<br>• Short term<br>• Long term<br>• Weight<br>• Breastfeeding<br><br>What effect, if any, lifestyle might have on your health?<br>• How does that affect your health now?<br>• How does that affect your health in the long term?<br>• How about the effect on weight?
Lifestyle and health consequences: Child<br>What is your greatest health concern for your child at the moment?<br>• Weight<br>• Breastfeeding<br><br>What effect, if any, your lifestyle might have on your child's/children's health?<br>• Follow up on the greatest health concern mentioned
Healthy lifestyle practices

What are some of the things you are doing now that contribute to a healthy lifestyle for yourself?

- Sleep
- Stress
- Diet
- Exercise
- Other

D) Facilitators and barriers

Facilitators

You mentioned some of the things you are doing for yourself to have healthy lifestyle, what helps you do these things?

- Friends
- Family
- Work arrangement
- Finances
- Geographical access
- Health professionals
- Community eg gym, mothers' group

Barriers

What are some of the things that prevent you from having a healthy lifestyle?

- Infant care
- Breastfeeding
- Friends
- Family
- Work arrangement
- Finances
- Geographical access

Opportunities

Were you able to overcome these barriers, if so how?

- Friends
- Family
- Work arrangement
- Finances
- Geographical access
- Health professionals
- Community eg gym, mothers' group

Co-parenting

How does your partner's involvement in parenting affect your ability to manage your lifestyle?

E) Preferred intervention

Who

Who are the best people to support you in having healthy lifestyle?

- Family

- Friends
- Health professionals
- Community

What

What do you need most to improve your lifestyle?

- Health information—mum or baby
- Coaching—set goals, plans, feedback and accountability
- Strategies, ideas

Where

Where would you prefer to attend this service?

- GP
- MCH
- Online
- Community setting
- Allied health (eg dietitian, EP, psychologist, physiotherapist)

When

Thinking back from the time you gave birth, when is the best time to start having this support?

- Early or late postpartum

Dose

How intensely would you like to be supported?

- Duration
- Frequency
- Self-guided

Cost

How willing are you to pay for this service?

- Would paying help motivate you?

F) Future recommendation

If anything is possible, what services/information/ resources would help postpartum women to have healthier lifestyles?

What are your thoughts on mum's coaching each other? Would you be interested to be involved if given the opportunity? Would you expect to be paid?

G) Thank the participant. Snowball recruit and further research

This is the end of the interview. Thank you for your time. Do you have any friends that may be interested in being interviewed? If they are interested in the study, you could pass on my contact details to them.
We may have further work in this area, such as getting all the interviewed mums together to plan the next steps. Would you like be contacted, so you can decide if you would like to participate then?
